# Prognostic factors for long-term survival in anti-MDA5–positive dermatomyositis: a 10-year cohort study

**DOI:** 10.3389/fmed.2026.1908926

**Published:** 2026-10-08

**Authors:** Yan Wang, Xueliang Zhang, Xiaolei Ma, Xin Zhang, Sheng-Ming Dai, Yin Liu, Shengnan Zhao, Zhiyong Chen

**Affiliations:** 1 Department of Rheumatology and Immunology, The Second People’s Hospital of Changzhou, The Third Affiliated Hospital of Nanjing Medical University, Changzhou, China; 2 Department of Rheumatology & Immunology, Arthritis Clinical Diagnosis and Treatment Centre, Shanghai Sixth People’s Hospital Affiliated to Shanghai Jiao Tong University School of Medicine, Shanghai, China; 3 Department of Rheumatology and Immunology, The Affiliated Drum Tower Hospital, Nanjing University Medical School, Nanjing, China; 4 Department of Respiratory and Critical Care Medicine, The Affiliated Drum Tower Hospital, Nanjing University Medical School, Nanjing, China

**Keywords:** anti-MDA5 positive dermatomyositis, interstitial lung disease, LASSO regression, long-term survival, prognostic factors

## Abstract

**Background:**

Anti-melanoma differentiation-associated gene 5-positive dermatomyositis (anti-MDA5+ DM) is a severe subtype frequently complicated by rapidly progressive interstitial lung disease (RP-ILD), leading to high early mortality. However, factors associated with long-term survival beyond 10 years remain largely uncharacterized. This exploratory analysis aimed to identify baseline factors associated with long-term all-cause mortality in a 10-year follow-up cohort using LASSO-Cox regression.

**Methods:**

This retrospective longitudinal study enrolled 84 inpatients diagnosed with anti-MDA5+ DM between 2010 and June 2014; surviving patients had more than 10 years of potential follow-up until March 2026. The primary endpoint was all-cause mortality. All candidate covariates, including clinical features, laboratory parameters, HRCT score, and initial treatment variables, were entered simultaneously into LASSO-penalized Cox regression with 10-fold cross-validation, and retained variables were directly entered into a multivariable Cox model. The proportional hazards assumption was tested, and bootstrap resampling was performed to assess selection stability. Survival was illustrated using Kaplan–Meier curves with log-rank tests.

**Results:**

Among the 84 patients (27 males and 57 females), 41 (48.8%) were alive at the final follow-up and 43 (51.2%) had died. As a descriptive observation, 25 surviving patients (29.8% of the total cohort) had maintained sustained drug-free remission for over 2 years. Four baseline factors were independently associated with long-term mortality. Older age (HR = 1.05 per 1-year increase, *P* < 0.001), fever on admission (HR = 2.08, *P* = 0.036), and higher HRCT score (HR = 1.94 per 1-point increase, *P* < 0.001) were associated with higher mortality, whereas concurrent arthritis was associated with lower mortality (HR = 0.45, *P* = 0.022). Male sex showed a trend toward higher mortality that was not statistically significant after adjustment (*P* = 0.086).

**Conclusion:**

Older age, admission fever, higher HRCT score, and absence of arthritis — all assessable at admission — were independently associated with all-cause mortality over long-term follow-up in the full anti-MDA5+ DM cohort; landmark analysis showed only arthritis and HRCT score remained associated with mortality after year 1. Given the limited sample size and lack of external validation, these exploratory findings are hypothesis-generating and require confirmation in independent multicenter cohorts.

## Introduction

Anti-melanoma differentiation-associated gene 5-positive dermatomyositis (anti-MDA5+ DM) is a subtype of DM that frequently complicates with rapidly progressive interstitial lung disease (RP-ILD). Anti-MDA5+ DM-RP-ILD is relatively common in East Asian populations and is associated with extremely high early mortality (1). Our previous study demonstrated that these patients have a dismal short-term prognosis, with a six-month survival rate of only 41% (2).

To date, the vast majority of clinical investigations on anti-MDA5+ DM have focused on predicting RP-ILD and mortality risk, as well as exploring intensive treatment regimens, with several risk prediction models and intensive therapeutic protocols having been developed (3). In recent years, long-term follow-up studies have revealed that many patients who survive the initial 1–2 years achieve sustained disease stability with a relatively low late mortality rate; however, the factors associated with long-term remission in MDA5 + dermatomyositis remain poorly understood (4). Given that prolonged intensive immunosuppressive therapy carries significant risks of infection and malignancy (5), identifying baseline factors associated with long-term survival is clinically relevant for understanding disease trajectories and informing future studies of individualized management.

The aim of the present study was to identify baseline factors associated with long-term all-cause mortality in a single-center cohort of patients with anti-MDA5+ DM, in which surviving patients had more than 10 years of potential follow-up. Given the limited sample size and retrospective design, this study was conceived as an exploratory analysis of prognostic factors rather than the development of a validated prediction model.

## Methods

### Study population

This retrospective longitudinal follow-up study screened inpatients diagnosed with anti-MDA5-positive dermatomyositis between 2010 and June 2014 at the Department of Rheumatology and Immunology, the Affiliated Drum Tower Hospital, Nanjing University Medical School. The present study included the 104 patients managed by the corresponding author's (Z.C.) team, whose complete baseline data were accessible; this cohort therefore overlaps with, but is not identical to, the population reported in our previous report (2). Of these 104 patients, 20 (19.2%) were lost to follow-up and excluded, yielding the final cohort of 84 patients (Supplementary Figure S1). All patients initially met the classic dermatomyositis diagnostic criteria proposed by Bohan and Peter, or the classification criteria for clinically amyopathic dermatomyositis (2). All 84 patients had interstitial lung disease on HRCT, as anti-MDA5 testing was performed almost exclusively in patients with ILD during that era. RP-ILD status could not be reliably adjudicated retrospectively because dyspnea documentation, pulmonary function tests, and serial HRCT images were incompletely available. As all patients were enrolled prior to the commercial availability of anti-MDA5 antibody testing in China, serum anti-MDA5 antibody was measured using validated methods previously described in our previous publication (2). Patients were followed up through telephone contact, outpatient visits, or hospital record inquiries. Follow-up duration was defined as the interval from the date of anti-MDA5+ DM diagnosis to the date of death or last follow-up visit (March 2026). The study protocol, including the retrospective analysis of clinical data and the telephone follow-up procedure, was approved by the Ethics Committee of the Affiliated Drum Tower Hospital, Nanjing University Medical School (approval No. 2025-0768-01; approved on September 5, 2025). Given the retrospective, non-interventional design, the requirement for individual written informed consent was waived by the Ethics Committee. Telephone follow-up was limited to ascertaining survival status and current medication use; no additional laboratory or imaging data were collected through telephone contact. All data were de-identified prior to analysis, and patient confidentiality was strictly maintained throughout the study.

### Data collection

Detailed baseline clinical and laboratory data of all enrolled patients at admission were collected comprehensively. Clinical baseline indicators included general demographic information (age, sex), typical clinical symptoms (fever, skin rash, muscle weakness, arthritis, interstitial lung disease, etc.), and basic disease characteristics. Laboratory indicators covered routine blood tests, inflammatory indicators, immune antibody spectrum, liver and kidney function, muscle enzyme levels, and other serological examination results. The presence of ILD was determined by high-resolution computed tomography (HRCT) findings. The extent of pulmonary involvement on HRCT was evaluated using a semi-quantitative scoring system (1 = <10%, 2 = 10%–25%, 3 = 25%–50%, 4 = >50% of the involved area), yielding a total score ranging from 1 to 4, with higher scores indicating more extensive pulmonary involvement. The HRCT images were independently reviewed by an experienced rheumatologist (Z.C.) and an experienced pulmonologist (Y.L.), with disagreements resolved by consensus. The long-term survivors were followed up continuously for more than 10 years. The survival status, time and cause of death were recorded in detail.

### Statistical analysis

All statistical analyses were performed using R software (version 4.2.1). Baseline characteristics were compared between survivors and non-survivors stratified by all-cause mortality. Categorical variables including Sex, Fever, Arthritis, CTX, HCQ and other immunosuppressants (Other IS) were assessed using the chi-square test or Fisher's exact test as appropriate. Continuous variables including Age, Onset-diagnosis time, HRCT score, WBC, Neu, Lym, Hb, PLT, AST, TG, CK, LDH, ESR, CRP, ferritin and prednisone dose were compared with Student's t-test for normally distributed data or the Mann–Whitney U test for non-normally distributed data. A two-sided *P* < 0.05 was defined as statistically significant. Based on clinical background, key clinically relevant variables were predefined and included as candidate predictors before dimensionality reduction.

LASSO Cox regression with 10-fold cross-validation was applied to reduce dimensionality and avoid overfitting, using the “*glmnet*” package in R. We did not set penalty factors to force the retention of predefined core clinical variables during LASSO dimensionality reduction. The optimal penalty parameter (*λ*) was determined according to the minimum partial likelihood deviance criterion (lambda.min). Bootstrap resampling with 100 replicates was conducted to assess the stability of LASSO variable selection: the complete LASSO cross-validation pipeline was repeated on each resampled cohort, and the selection frequency of each candidate variable across all replicates was calculated and visualized.

All variables retaining non-zero regression coefficients from LASSO screening were simultaneously entered into multivariate Cox regression without stepwise elimination to identify independent prognostic factors, using the *survival* package. The Schoenfeld residual test was carried out to verify the proportional hazards (PH) assumption of the multivariate Cox model, eliminating bias derived from time-varying hazard ratios. To test potential nonlinear associations between continuous predictors and mortality risk, separate Cox models incorporating linear and quadratic terms of age and HRCT score were constructed.

A primary multivariate Cox model containing five core clinical predictors was established for quantification of independent prognostic effects. Sensitivity analysis was performed by removing the only marginally non-significant variable (sex) to confirm consistent prognostic effects of the remaining predictors. To further examine the temporal stability of the prognostic associations and to assess the potential dominance of early mortality, a landmark analysis with a 12-month cutoff was conducted among patients who survived no less than 12 months after disease onset, with the identical set of five predictors refitted in the landmark Cox model.

Optimal cut-off values for continuous predictors in survival stratification were calculated using the *survminer* package. Kaplan–Meier survival curves paired with log-rank tests were generated for survival visualization. All figures, including cross-validation curves, LASSO coefficient profiles, bootstrap variable selection frequency plots, and overall survival curves, were plotted with the *ggplot2* package. A two-sided *P*-value < 0.05 was defined as statistically significant.

## Results

### Clinical characteristics of patients with MDA5 + DM

A total of 84 patients with anti-MDA5-positive DM were enrolled in the present study, including 27 males and 57 females. Baseline demographic, clinical, radiological, laboratory and initial treatment variables stratified by survival outcome are listed in Table 1. All patients were followed until death or March 2026, whichever came first; surviving patients had at least 10 years of potential follow-up. The median follow-up duration, estimated by the reverse Kaplan–Meier method, was 154 months. The cumulative mortality was 34.5% at 6 months, 40.5% at 1 year, 51.2% at 5 years, and 51.2% at 10 years, with almost no additional deaths after 5 years. At the final follow-up visit, 41 patients were alive, while 43 patients had died, resulting in an overall all-cause mortality of 51.2% (43/84).

**Table 1:** Clinical characteristics and outcomes in patients with anti-MDA5 + dermatomyositis.

Parameter	Long-term survivor (*n* = 41)	Dead (*n* = 43)	*P* value
Sex (Male, %)	8 (19.5%)	19 (44.2%)	0.029
Age (years)	46.3 ± 11.6	53.2 ± 10.8	0.006
Onset-diagnosis time (months)	2.60 (2.47)	3.80 (3.76)	0.085
Fever (%)	21 (51.2%)	31 (72.1%)	0.081
Arthritis (%)	27 (65.9%)	14 (32.6%)	0.005
HRCT score	1.98 (0.79)	2.81 (0.85)	<0.001
Laboratory findings			
WBC ( × 10⁹/L)	5.38 (2.08)	5.53 (2.94)	0.793
Neu (×10⁹/L)	4.14 (1.94)	5.78 (9.29)	0.264
Lym (×10⁹/L)	0.86 (0.41)	1.36 (3.99)	0.424
Hb (g/L)	125 (13.7)	130 (16.4)	0.168
PLT (×10⁹/L)	168 (50.5)	172 (59.5)	0.749
AST (U/L)	96.7 (104)	158 (226)	0.112
TG (mmol/L)	1.87 (1.17)	1.77 (0.99)	0.678
CK (U/L)	269 (578)	132 (140)	0.147
LDH (U/L)	450 (250)	611 (494)	0.063
ESR (mm/h)	33.1 (19.8)	40.6 (19.1)	0.082
CRP (mg/L)	8.47 (16.0)	13.6 (19.8)	0.192
Ferritin (ng/mL)	919 (780)	1,794 (2,304)	0.022
Initial Treatments			
Prednisone (mg/d)	72.4 (78.9)	91.2 (92.5)	0.318
CTX (%)	23 (56.1%)	22 (51.2%)	0.815
HCQ (%)	26 (63.4%)	22 (51.2%)	0.361
Other IS (%)	22 (53.7%)	24 (55.8%)	1.000

*Values are presented as mean ± SD or median (interquartile range) as appropriate based on normality tests, or n (%).*

MDA5, melanoma differentiation-associated gene 5; HRCT, high resolution CT; WBC, white blood cell; Neu, neutrophil count; Lym, lymphocyte count; Hb, hemoglobin; PLT, platelet count; AST, aspartate aminotransferase; TG, triglyceride; CK, creatine kinase; LDH, lactate dehydrogenase; ESR, erythrocyte sedimentation rate; CRP, C-reactive protein. CTX, cyclophosphamide; HCQ, hydroxychloroquine; Other IS, other immunosuppressants (including tacrolimus, cyclosporine, etc.).

Notably, among the surviving cohort, 25 patients (29.8% of the total population) sustained complete cessation of all DM-related medications (including anti-fibrotic agents) for more than 2 consecutive years without re-initiation of therapy, a status hereafter referred to as sustained drug-free remission. Compared with surviving patients, non-survivors presented a significantly higher proportion of male sex (44.2% vs. 19.5%, *P* = 0.029) and older baseline age [53.2 (10.8) vs. 46.3 (11.6) years, *P* = 0.006]. The prevalence of concomitant arthritis was markedly lower in the death group than in the remission group (32.6% vs. 65.9%, *P* = 0.005). Besides, non-survivors had significantly higher baseline HRCT scores for ILD [2.81 (0.85) vs. 1.98 (0.79), *P* < 0.001]. For laboratory indices, serum ferritin level was significantly elevated among non-survivors [1,794 (2,304) vs. 919 (780) ng/mL, *P* = 0.022]. Other indicators including onset-to-diagnosis interval, fever incidence and routine blood/biochemical test results showed no statistically significant intergroup differences, as did all initial immunosuppressive treatment regimens (all *P* > 0.05).

### LASSO regression for candidate variable screening

LASSO regression with 10-fold cross-validation was implemented to further compress candidate prognostic variables, without setting penalty factors to forcibly retain core clinical predictors (Figures 1A,B). To prevent model overfitting and retain clinically relevant predictors, the optimal *λ* value was determined by the minimum partial likelihood deviance criterion rather than the one-standard-error rule. At the optimal log(*λ*) cutoff, only five variables maintained non-zero regression coefficients and were retained for subsequent multivariate Cox modeling: sex, age, admission fever, concomitant arthritis, and HRCT score. All remaining laboratory and clinical indicators including neutrophil count, Hb, AST, LDH, ferritin and immunosuppressive treatments were eliminated as their coefficients shrank to zero under the optimal regularization penalty. Bootstrap resampling further supported the stability of these five predictors, which all achieved selection frequencies over 50% across 100 replicates (Supplementary Figure S2).

**Figure 1:**
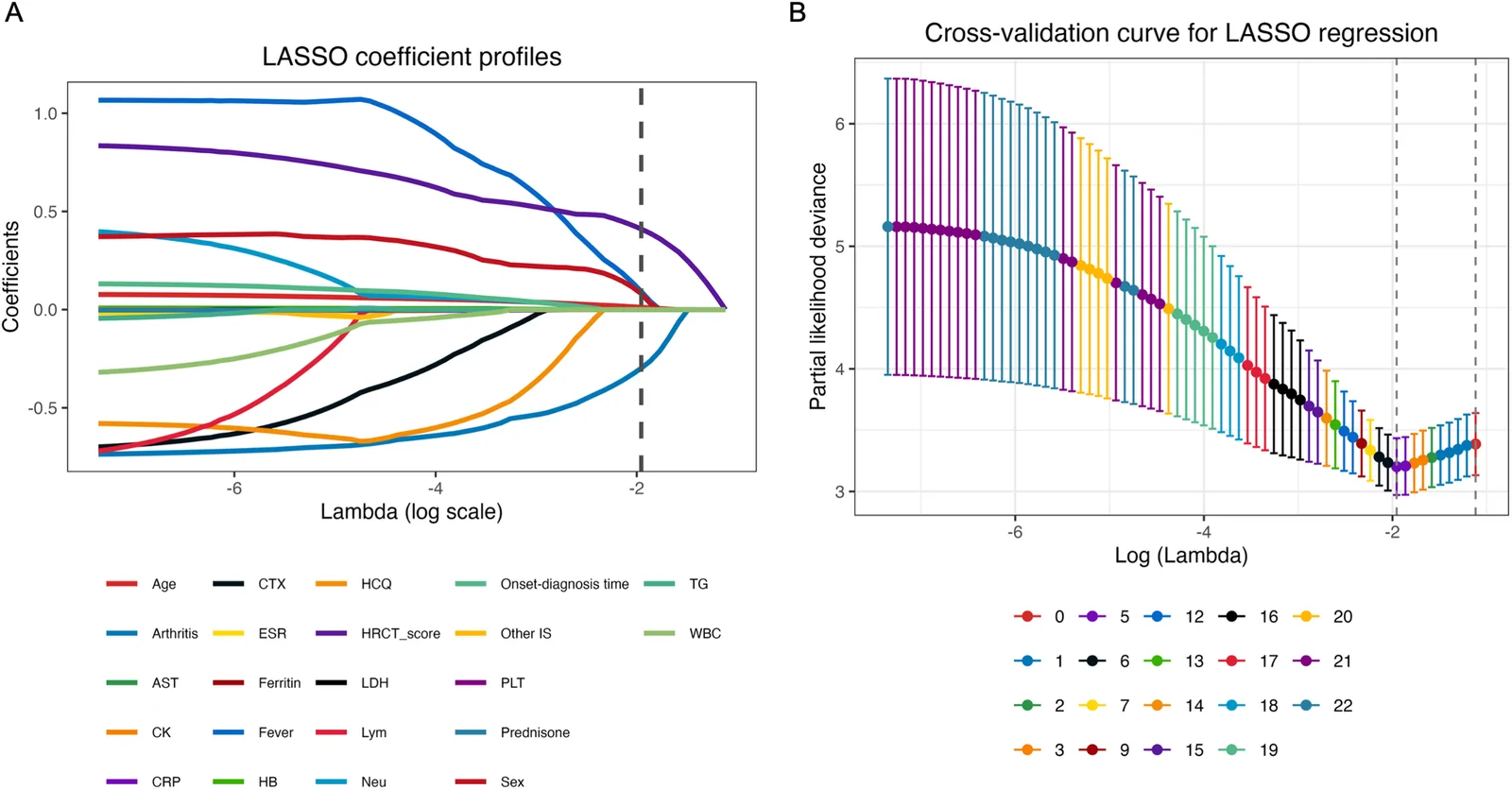
Variable selection for model development using least absolute shrinkage and selection operator (LASSO) regression. **(A)** LASSO coefficient profiles of candidate variables. **(B)** Cross-validation curve for LASSO regression. Five variables were selected using LASSO regression analysis. WBC, white blood cell; Neu, neutrophil count; Lym, lymphocyte count; Hb, hemoglobin; PLT, platelet count; AST, aspartate aminotransferase; TG, triglyceride; CK, creatine kinase; LDH, lactate dehydrogenase; ESR, erythrocyte sedimentation rate; CRP, C-reactive protein, CTX, cyclophosphamide; HCQ, hydroxychloroquine; Other IS, other immunosuppressants (including tacrolimus, cyclosporine, etc.).

### Quantification of independent prognostic effects using multivariate Cox regression

Following variable screening via LASSO Cox regularization, the five predictors retained after penalization (sex, age, admission fever, concomitant arthritis, HRCT score) were simultaneously incorporated into multivariate Cox proportional hazards regression without stepwise elimination. Schoenfeld residual testing confirmed all variables satisfied the proportional hazards assumption (all *P* > 0.05, Supplementary Figure S3). Separate Cox models with quadratic terms of age and HRCT score revealed no obvious nonlinear associations between the two continuous variables and all-cause mortality risk. The finalized multivariate Cox regression identified four of the five screened variables as factors independently associated with all-cause mortality in anti-MDA5-positive dermatomyositis patients (Figure 2). In the final multivariable Cox model, each 1-year increase in baseline age was independently associated with a higher mortality hazard (HR = 1.05, 95% CI: 1.02–1.09, *P* < 0.001). Patients presenting with fever at admission had a higher all-cause mortality risk (HR = 2.08, 95% CI: 1.05–4.14, *P* = 0.036), and each 1-point increase in HRCT score was independently associated with higher long-term mortality (HR = 1.94, 95% CI: 1.35–2.79, *P* < 0.001). In contrast, concurrent arthritis was independently associated with a lower risk of death (HR = 0.45, 95% CI: 0.22–0.89, *P* = 0.022). Male sex showed a trend toward higher mortality, but the association did not reach statistical significance after adjustment for HRCT score and other covariates (HR = 1.79, 95% CI: 0.92–3.46, *P* = 0.086). A sensitivity analysis excluding sex yielded essentially unchanged effect estimates for the remaining four variables, supporting the stability of these exploratory prognostic factors.

**Figure 2:**
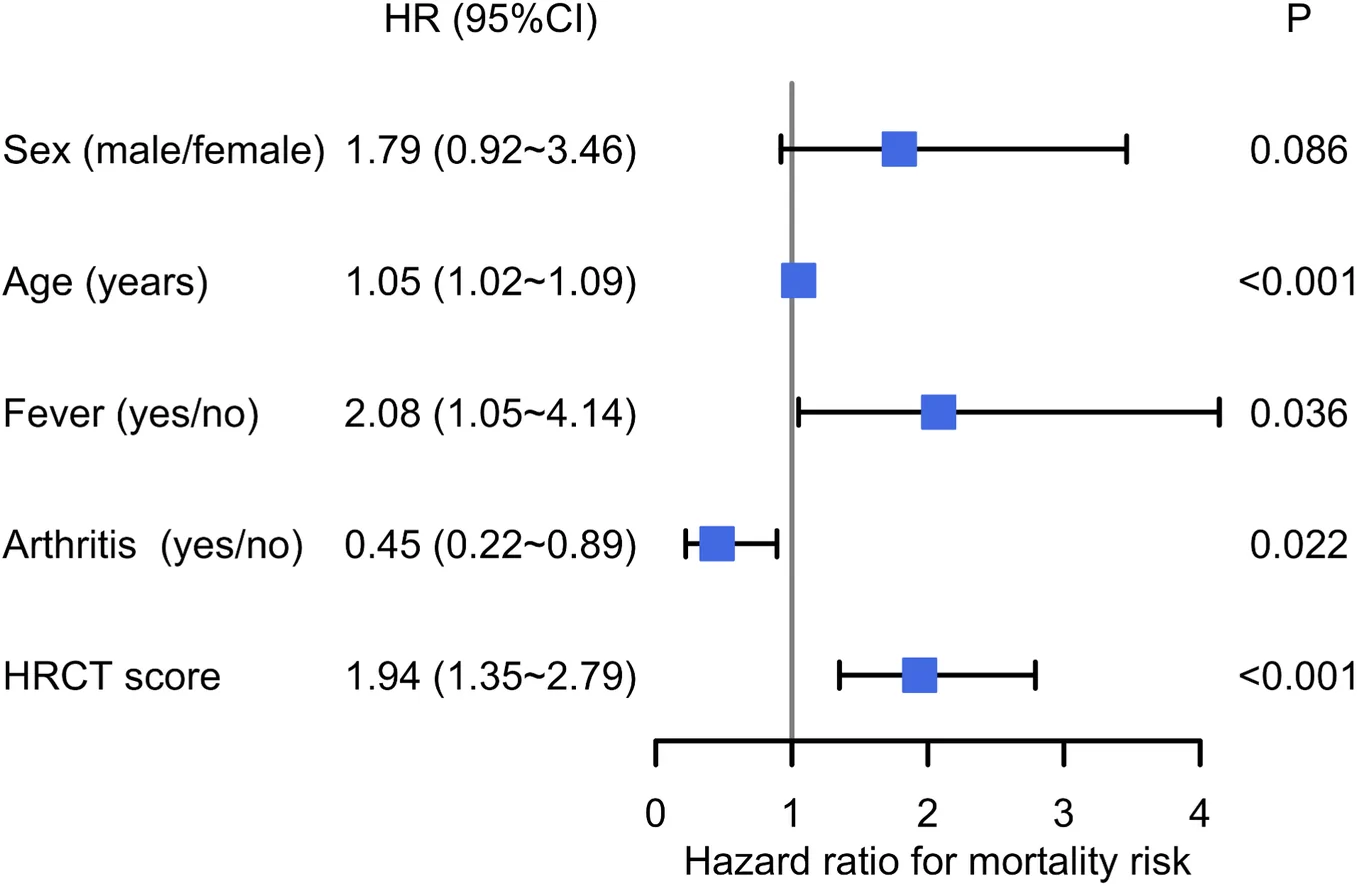
Multivariate Cox regression forest plot showing hazard ratios of the LASSO-retained variables for mortality among anti-MDA5 + dermatomyositis patients. Squares and horizontal lines correspond to hazard ratio (HR) values and their 95% confidence intervals (CI), respectively; the vertical line at hazard ratio = 1 is the reference line for statistical significance.

### Temporal prognostic characteristics of core variables assessed by landmark analysis

To further eliminate the confounding influence of early mortality and temporally examine the stability of prognostic variables, we performed a 12-month landmark Cox regression analysis. Of the total 84 patients in the original cohort, 34 patients died within the first 12 months and none were censored before 12 months; therefore, 50 patients who survived more than one year after disease onset were included in the landmark cohort (Table 2). In the landmark analysis, concomitant arthritis and HRCT score remained independent prognostic factors for long-term all-cause mortality, whereas advanced age did not (HR = 1.08 per 1-year increase, 95% CI: 0.98–1.18, *P* = 0.13). The attenuation of the age effect may reflect limited statistical power due to the halved sample size and few events beyond 12 months rather than a true absence of long-term effect, although an early-concentrated effect cannot be excluded. Arthritis remained independently associated with a lower risk of death (HR = 0.10, 95% CI: 0.02–0.55, *P* = 0.01), and a higher HRCT score remained associated with long-term mortality (HR = 2.57 per 1-point increase, 95% CI: 1.17–5.62, *P* = 0.02). In contrast, admission fever and male sex were no longer significantly associated with mortality among 1-year survivors. This pattern is consistent with the predominance of early deaths in this cohort and may indicate that the associations of fever and sex with mortality are driven mainly by events within the first year of the disease course; however, the reduced sample size of the landmark subgroup limits statistical power, and formal testing of time-varying effects would be required to confirm true time-dependence. Taken together, arthritis and HRCT score showed associations with mortality that persisted beyond the early high-risk period, whereas the associations of fever, sex and possibly age appeared largely confined to early mortality within the first year.

**Table 2:** Landmark multivariate Cox regression analysis in patients surviving ≥12 months (*n* = 50).

Variable	Regression coefficient (B)	Wald *χ*^2^	Hazard Ratio (HR)	95% Confidence Interval	*P*-value
Sex (Male)	0.27	0.08	1.31	0.20–8.44	0.78
Age	0.07	2.25	1.08	0.98–1.18	0.13
Fever	0.4	0.3	1.49	0.36–6.15	0.58
Arthritis	−2.34	6.94	0.10	0.02–0.55	**0**.**01**
HRCT score	0.94	5.54	2.57	1.17–5.62	**0**.**02**

Two-sided *P* < 0.05 was considered statistically significant; significant *P* values are bolded. HRCT, high resolution CT; HR, hazard ratio; CI, confidence interval. All numerical values were rounded to two decimal places.

### Kaplan–Meier survival analysis stratified by candidate prognostic factors

Kaplan–Meier survival analysis with log-rank test was used to illustrate survival differences across strata of the variables retained in the multivariable Cox model; these unadjusted analyses are presented for visualization and do not constitute model validation. Total Kaplan–Meier survival curves were shown in Figure 3A. Male patients showed lower long-term survival than female patients (Figure 3B, log-rank *P* = 0.007); notably, the association of sex was attenuated and non-significant in the multivariable model, suggesting that it is partly explained by differences in pulmonary severity. Patients older than 50 years had lower survival probability than younger patients (Figure 3C, log-rank *P* < 0.001); age was dichotomized at 50 years for visualization only and was analyzed as a continuous variable in all regression models. Patients with arthritis showed higher survival than those without (Figure 3D, log-rank *P* = 0.0033), and patients with fever at admission showed lower survival than afebrile patients (Figure 3E, log-rank *P* = 0.029). Patients with an HRCT score > 2 showed lower overall survival than those with a score ≤ 2 (Figure 3F, log-rank *P* < 0.001); this cut-off was chosen for exploratory visualization

**Figure 3:**
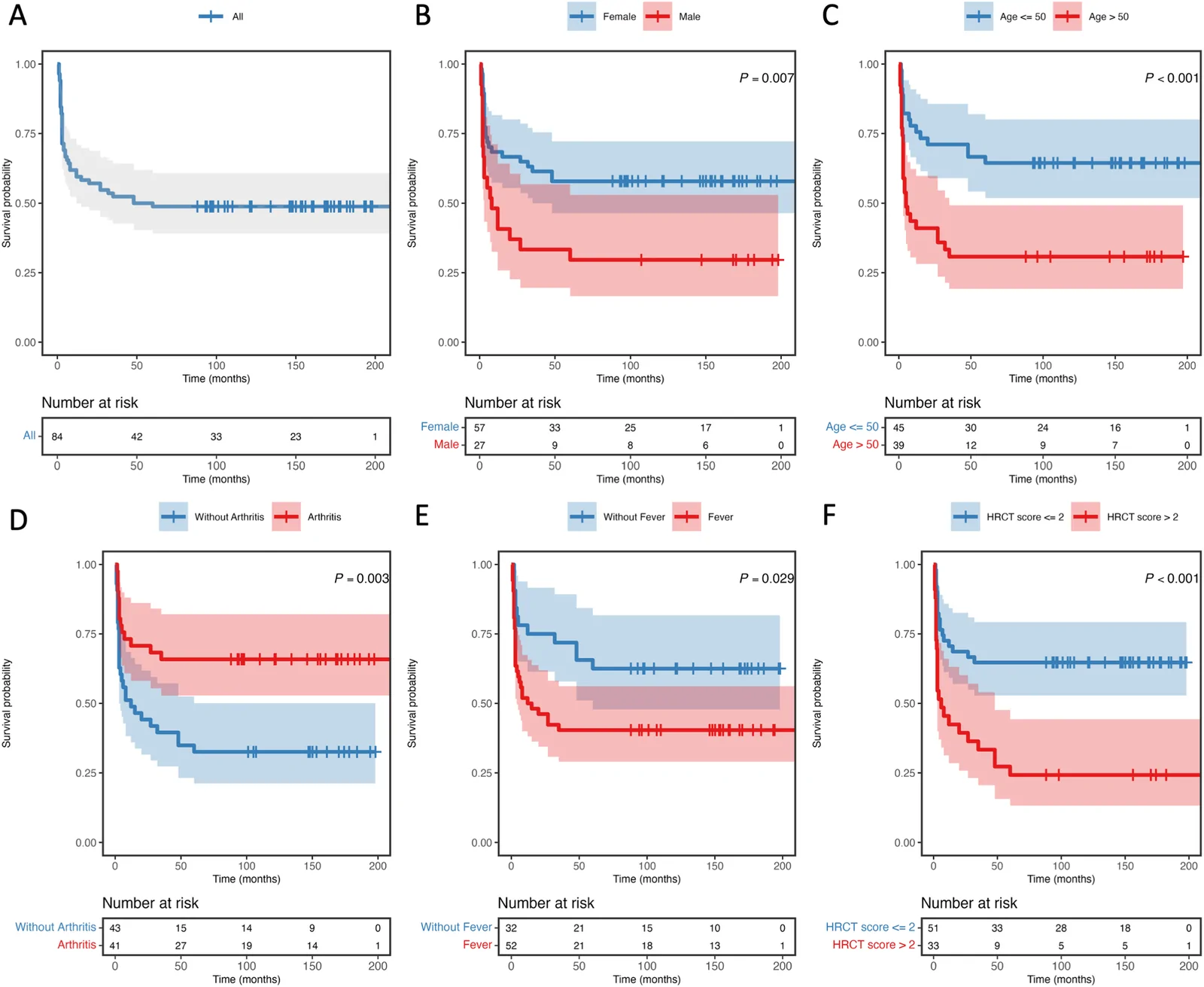
Kaplan–Meier survival curves for all-cause mortality stratified by the five LASSO-retained variables in patients with anti-MDA5 + dermatomyositis. **(A)**Total Kaplan–Meier survival curves; **(B)** sex; **(C)** age (<50 vs. ≥ 50 years); **(D)** concomitant arthritis; **(E)** baseline fever status; **(F)** HRCT score (≤2 vs. > 2). Log-rank *P* values are displayed in each subpanel, and shaded regions indicate the 95% confidence interval of survival estimates.

## Discussion

In this longitudinal cohort study in which surviving patients had more than 10 years of potential follow-up, 48.8% of patients with anti-MDA5+ DM were alive at the end of follow-up; as a descriptive observation, 29.8% of the total cohort had sustained drug-free remission. In contrast to the majority of prior studies that have focused on RP-ILD and short-term mortality risk, the present study explored factors associated with all-cause mortality over long-term follow-up. In the full cohort, older age (modeled as a continuous variable), admission fever, higher HRCT score, and the absence of arthritis were independently associated with higher mortality, while only arthritis and HRCT score retained prognostic value beyond the first year. (Figure 3).

This finding is consistent with recent reports indicating that the 5-year relapse-free survival rate exceeds 77% among patients who survive the induction phase (4, 6). A plausible explanation is spontaneous immune resetting following intensive immunosuppression — that is, transient autoimmunity does not necessarily imply lifelong treatment dependence.

The adverse prognostic impact of older age and male sex observed in this cohort is consistent with findings from recent large-scale studies. In the present cohort, non-survivors were significantly older than survivors (53.2 ± 10.8 years vs. 46.3 ± 11.6 years) and had a higher proportion of males (44.2% vs. 19.5%). A meta-analysis encompassing 15 studies and 1,153 patients confirmed that older age is a significant risk factor for mortality (HR = 1.04) (3). Similarly, the FLATCAN model developed by Zong et al., which enrolled 621 patients, identified age at disease onset as one of seven key mortality predictors (7). Consistent with these observations, each 1-year increase in baseline age was independently associated with a 5% increase in the mortality hazard in our full cohort (HR = 1.05, 95% CI: 1.02–1.09). This association was no longer significant among patients surviving beyond 12 months, suggesting it was mainly driven by early mortality. This association may reflect age-related decline in immune reserve and regenerative capacity. Male sex was associated with higher mortality in unadjusted analysis, but the association was attenuated and no longer statistically significant after adjustment for HRCT score and other covariates (HR = 1.79, 95% CI: 0.92–3.46, *P* = 0.086), suggesting that the higher mortality observed in male patients may be partly mediated by greater pulmonary involvement rather than by sex *per se*; sex-related differences in interferon signaling remain a hypothesis warranting mechanistic study (8).

In our cohort, admission fever was independently associated with higher mortality (HR = 2.08, 95% CI: 1.05–4.14, *P* = 0.036). Fever was present at baseline in 72.1% of non-survivors compared with 51.2% of survivors, and this association was not significant in the 12-month landmark cohort. This finding is consistent with a recent meta-analysis by Yang et al., which identified fever as a significant mortality predictor (HR = 2.56) (3), and a study of 71 patients with anti-MDA5+ DM-ILD reported fever rates of 80% among non-survivors versus 36.96% among survivors (*P* < 0.001) (9). Fever may reflect a highly inflammatory phenotype characterized by increased cytokine production, potentially overlapping with the “vasculopathic subtype” described in recent clinical classifications (10). It should be noted that fever, CRP, ESR, and ferritin are inter-correlated markers of systemic inflammatory burden; the retention of fever by LASSO likely reflects the selection of one representative marker among correlated variables, rather than indicating that CRP, ESR, or ferritin lack prognostic relevance. From a practical standpoint, fever is readily assessable at the bedside and may serve as an early clue prompting evaluation for RP-ILD or occult infection (11).

An intriguing observation of our study is that concurrent arthritis was independently associated with lower long-term mortality (HR = 0.45, 95% CI: 0.22–0.89, *P* = 0.022). Patients with baseline arthritis had markedly lower mortality than those without (32.6% vs. 65.9%). This observation is supported by recent cluster analyses of anti-MDA5+ DM patients. Zong et al. identified three distinct clinical subgroups, in which the absence of arthritis was a strong predictor of poor outcome; patients with neither Raynaud's phenomenon nor arthritis had the highest probability of belonging to the most unfavorable prognostic group (7). One hypothesis is that arthritis defines a distinct immune phenotype characterized by predominant joint involvement but less aggressive pulmonary or systemic vasculopathic disease. This is consistent with the proposed clinical classification of anti-MDA5+ DM into RP-ILD, rheumatic, and vasculopathic subtypes, in which the rheumatic subtype — characterized by arthritis — carries a more favorable prognosis (12).

Serum ferritin was not retained by LASSO-penalized regression, despite higher levels in non-survivors than survivors in unadjusted comparison [1,794 (2,304) vs. 919 (780) ng/mL, *P* = 0.022]. This may appear to contrast with several studies identifying ferritin as a mortality risk marker: the FLATCAN model included ferritin as a key variable (7), an Italian cohort reported higher ferritin in non-survivors (median 4,670 vs. 542.5 ng/mL, *P* = 0.019) (5), and a meta-analysis by Yang et al. found ferritin ≥ 800 ng/mL to be associated with increased mortality (HR = 6.17, 95% CI: 2.51–15.20) (3). However, ferritin, fever, CRP, and ESR are inter-correlated markers of inflammatory burden, and the retention of correlated variables by penalized regression is partly interchangeable. Moreover, as an acute-phase reactant, ferritin may normalize following successful immunosuppression and thus lose long-term prognostic value; consistently, in 38 of 41 surviving patients (92.7%), ferritin returned to normal within 6 months of treatment initiation. Ferritin may therefore be more informative for short-term than for long-term risk assessment.

Several limitations of this study warrant acknowledgment. First, the single-center, retrospective design may introduce selection bias; the exclusion of patients lost to follow-up may have introduced survivorship bias, and the relatively small sample size limits generalizability. External validation in independent, multicenter cohorts is therefore required. Second, this was an exploratory analysis of prognostic factors: no formal measures of discrimination or calibration were assessed, and no internal or external validation was performed; the identified factors therefore require confirmation in independent, multicenter cohorts before any clinical application. Third, initial treatment variables were included among candidate predictors in the LASSO screening but were not retained in the final multivariate Cox model. Therefore, residual confounding attributable to treatment intensity and changes in treatment strategies across the study period (2010–2026) cannot be excluded, and treatment-related adverse events were not systematically assessed. Fourth, because all patients were enrolled before 2014 and followed retrospectively, longitudinal biomarker data — including serial anti-MDA5 antibody titers, which correlate with relapse risk (13), as well as lymphocyte subset counts and KL-6 (14,15,16) — were not routinely available. Fifth, variables with substantial missing data, such as forced vital capacity and diffusing capacity for carbon monoxide, could not be included, precluding a competing-risk analysis of ILD-specific death; causes of death were ascertained retrospectively and misclassification cannot be excluded. Sixth, the HRCT score was based on a semi-quantitative, non-validated scoring system, and the image review was not performed in a blinded manner, potentially introducing measurement and inter-observer variability. Finally, sustained drug-free remission was reported descriptively and was not modeled as an outcome; its determinants and durability require dedicated prospective study with appropriate competing-risk frameworks.

## Conclusion

In this exploratory analysis of a single-center cohort with more than 10 years of potential follow-up, older age (analyzed as a continuous variable), fever on admission, higher HRCT score, and the absence of arthritis were independently associated with all-cause mortality across the entire follow-up period, whereas only arthritis and HRCT score remained associated with mortality after year 1. As a descriptive observation, a substantial proportion of survivors (29.8% of the total cohort) maintained sustained drug-free remission. These findings describe associations with survival rather than predictors of remission or treatment response, and require validation in larger, independent, multicenter cohorts before they can inform individualized clinical decision-making.

## Data Availability

The raw data supporting the conclusions of this article will be made available by the authors, without undue reservation.
